# Assessing the potential impact of transmission during prolonged viral shedding on the effect of lockdown relaxation on COVID-19

**DOI:** 10.1371/journal.pcbi.1008609

**Published:** 2021-01-29

**Authors:** Burcu Tepekule, Anthony Hauser, Viacheslav N. Kachalov, Sara Andresen, Thomas Scheier, Peter W. Schreiber, Huldrych F. Günthard, Roger D. Kouyos

**Affiliations:** 1 Division of Infectious Diseases and Hospital Epidemiology, University Hospital Zurich, University of Zurich, Switzerland; 2 Institute of Medical Virology, University of Zurich, Switzerland; 3 Institute of Social and Preventive Medicine, University of Bern, Bern, Switzerland; 4 Division of Infectious Diseases and Hospital Hygene, University Hospital Zurich, University of Zurich, Switzerland; University of Notre Dame, UNITED STATES

## Abstract

A key parameter in epidemiological modeling which characterizes the spread of an infectious disease is the generation time, or more generally the distribution of infectiousness as a function of time since infection. There is increasing evidence supporting a prolonged viral shedding window for COVID-19, but the transmissibility in this phase is unclear. Based on this, we develop a generalized Susceptible-Exposed-Infected-Resistant (SEIR) model including an additional compartment of chronically infected individuals who can stay infectious for a longer duration than the reported generation time, but with infectivity reduced to varying degrees. Using the incidence and fatality data from different countries, we first show that such an assumption also yields a plausible model in explaining the data observed prior to the easing of the lockdown measures (relaxation). We then test the predictive power of this model for different durations and levels of prolonged infectiousness using the incidence data after the introduction of relaxation in Switzerland, and compare it with a model without the chronically infected population to represent the models conventionally used. We show that in case of a gradual easing on the lockdown measures, the predictions of the model including the chronically infected population vary considerably from those obtained under a model in which prolonged infectiousness is not taken into account. Although the existence of a chronically infected population still remains largely hypothetical, we believe that our results provide tentative evidence to consider a chronically infected population as an alternative modeling approach to better interpret the transmission dynamics of COVID-19.

## Author summary

A key epidemiological variable characterizing the spread of an infectious disease is the generation time, defining the time between successive cases in a chain of transmission. Although there is increasing evidence supporting a prolonged viral shedding window for COVID-19, it is currently unclear to what extent prolonged transmission also occurs. Here we investigate the plausibility of a population of chronically infected individuals who can stay infectious for a longer duration than the reported generation time, but with infectivity reduced to varying degrees. By using the daily case and fatality data from various countries, we show that the existence of a chronically infected population is not a possibility that can be easily rejected from an epidemiological perspective. Moreover, in case of a gradual easing on the lockdown measures, the predictions of the model including the chronically infected population vary considerably from the predictions of the conventional epidemiological models. Although it is not possible to either prove or disprove the existence of a hypothetical population purely by modeling, our results provide tentative evidence to consider a chronically infected population as an alternative modeling approach in assessing the transmission dynamics of COVID-19.

## Introduction

Mathematical models have been extensively used to understand the epidemic characteristics of oubreaks, in predicting future outcomes, and in shaping the national responses regarding control measures [1, 2]. Despite the time pressure, a considerable amount of work has been dedicated to modeling the pandemic of novel coronavirus (SARS-CoV-2) infections that began in China in late 2019 [3–6]. Although most of these studies are based on existing epidemic models such as SIR and SEIR-models, several features of the COVID-19 pandemic have been independently explored, leading to different generalizations of similar dynamical models. On one hand, having a variety of models is central to get a notion of the model sensitivity, on the other, it shows that different assumptions are equally favorable to explain the observed data given the right set of parameter choices, whereas they might lead to different projections on how the epidemic would follow in the future [7, 8]. This variability in future projections becomes especially important when a perturbation, such as the imposition or release of the control measures, is introduced to the dynamical system.

A key epidemiologic variable that characterizes the spread of an infectious disease is the generation time [9], i.e., the time between successive cases in a chain of transmission. Li *et al*. [10] estimated the generation time distribution to have a mean of 7.5 (95%CI 5.5–19) days based on 6 observations, whereas Ganyani *et al*. estimated the generation time distribution to have a mean of 5.20 (95%CI 3.78–6.78) days for Singapore and 3.95 (95%CI 3.01–4.91) days for Tianjin [11], Bi *et al*. estimated the generation time distribution to have a mean of 6.3 (95%CI 5.2–7.6) days [12], He *et al*. estimated the generation time distribution to have a mean of 5.8 (95%CI 4.8–6.8) days [13], and Hiroshi *et al*. estimated the generation time distribution to have a mean of 4.7 (95%CI 3.70–6.00) days. Considering all these studies, infectiousness is estimated to decline quickly within 4 to 8 days on average.

Additionally, certain cases of transmission arouse concern about prolonged shedding of SARS-CoV-2 after recovery [14]. Moreover, several studies show proof of active virus replication in upper respiratory tract tissues and prolonged viral shedding even after seroconversion for COVID-19, implying that the contagious period of COVID-19 might last more than one week after clinical recovery in a fraction of patients [15, 16]. De Chang *et al*. reported patients to be virus positive even after the resolution of symptoms up to 8 days [17]. Similarly, Young *et al*. reported a median duration of 12 days for viral shedding [18], and Zhou *et al*. observed a median duration of 20 days [19]. Tan *et al*. reported a special case where the duration of viral shedding persisted for 49 days from illness onset [20]. Such examples indicate an uncertainty regarding the skewness of the generation time distribution. In additon to this uncertainty, several studies estimating the generation time suffer from short follow-up times, selection bias, and recall bias, which might miss the individual cases with prolonged shedding durations. Considering that the duration of infectiousness is a critical parameter in dynamical models used for predictive purposes, it is important to consider the epidemiological plausibility of a a more heavy tailed generation time distribution than the reported distributions in the literature and investigate its impact on model outcomes.

To do so, we first develop a generalized SEIR model by segregating the infectious compartment into two as “primarily infected” and “chronically infected” population. We assume that primarily infected individuals have a higher infectiousnesss within the time window conventially considered as the generation time, during when they have the potential to develop symptoms and therefore be hospitalized. Afterwards, we assume that the non-hospitalized infecteds transition to the chronically infected phase before recovery and become less infectious, but may stay infectious for a longer duration. By doing so, we include the possibility of a prolonged viral shedding window in our model. Individuals in the chronically infected phase are relevant both for diagnosis (a positive test result) and disease transmission, and we will explore the role of both aspects in explaining the observed data.

Using the incidence and fatality data from different regions of Italy and different states of the U.S., we first show that our model is also a plausible candidate for explaining the data observed prior to the easing of the lockdown measures (relaxation) for a variety of combinations of prolonged duration and level of infectiousness assumed for the chronically infected population. Based on this conclusion, we test the predictive power of different models using the daily confirmed cases data after the introduction of relaxation in Switzerland, including a model without the chronically infected population to represent the models conventionally used. Only Swiss data is used to test the predictive power of different models due to the public avaliability of different data types (estimates on the effective reproductive number, the number of daily confirmed cases, daily deaths, hospitalized and ICU patients) in high temporal resolution. Our results show that, in case of a gradual easing on the lockdown measures, the predictions of the model including the chronically infected population vary considerably from those obtained under a model in which prolonged infectiousness is not taken into account. This variability is especially important when national policies on control measures are being formed, and also for the healthcare systems if projections such as the occupancy of the hospital ward or the ICU are calculated using similar dynamical models.

## Materials and methods

### Mathematical model

To describe the dynamics of the COVID-19 pandemic, we generalize the susceptible-exposed-infected-removed (SEIR) compartmental model by including eight different states denoted by *S*(*t*), *E*(*t*), *I*_*p*_(*t*), *I*_*c*_(*t*), *H*(*t*), *ICU*(*t*), *R*(*t*), and *X*(*t*), representing the number of susceptible individuals, exposed (infected but not yet infectious) individuals, primarily infected individuals, chronically infected individuals, hospitalized patients, patients in ICUs, recovered (immune) individuals, and deceased individuals at time *t*, respectively. To model the prolonged viral shedding in case of COVID-19, we segregate the infectious compartment into two by introducing two different compartments, namely the primarily infected (*I*_*p*_) and the chronically infected (*I*_*c*_) individuals. After the incubation period is complete, exposed individuals become primarily infected where they stay infectious within the reported duration of the infectious period of COVID-19. Conventionally, these individuals are assumed to stop being infectious and therefore stop contributing to the disease spread when the generation time is complete. Our purpose by including another step before recovery, i.e., the chronically infected compartment, is to model a scenario such that the primarily infected individuals transition to a state where they are less infectious but they may stay infectious and be diagnosed for a longer duration than the generation time, i.e. continue spreading the infection with reduced transmissibility.

**Fig 1:**
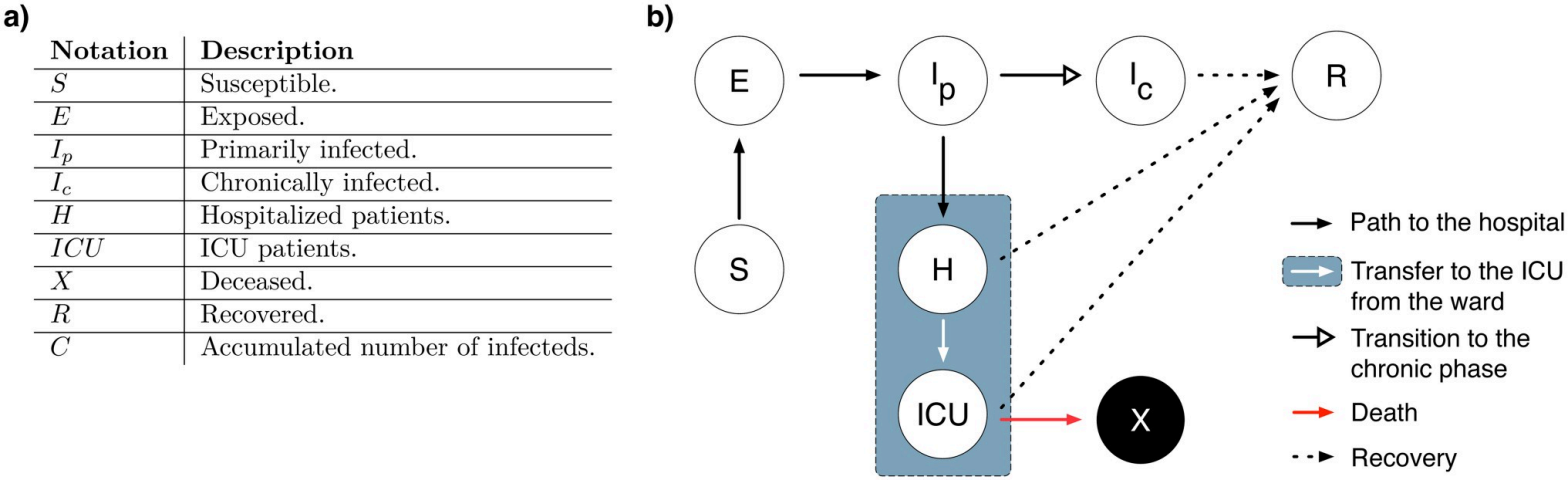
Illustration of the generalized SEIR model. **a)** Notation of the compartments and their corresponding descriptions. **b)** Schematic of the dynamical model given by Eq set 1.

Transitions between different compartments are illustrated in Fig 1, which can be translated into a system of ordinary differential equations, where each arrow, i.e., each process, is associated with a rate. This system is given by the Eq set 1, including the rates of processes as model parameters, and describes the rate of change of compartments over time. Model parameters are given in Table 1 with their corresponding descriptions and prior distributions. An additional compartment *C*(*t*) is included in the Eq set 1 to calculate the cumulative number of the positively diagnosed cases in the community, and does not play any role in the disease dynamics.

$$\begin{array}{c} \begin{array}{cc} \frac{dS(t)}{dt}= & -\frac{S}{N}(\beta_{p}I_{p}+\beta_{c}I_{c}),\\ \frac{dE(t)}{dt}= & +\frac{S}{N}(\beta_{p}I_{p}+\beta_{c}I_{c})-\tau E,\\ \frac{dI_{p}\left(t\right)}{dt}= & +\tau E-\gamma_{p}I_{p},\\ \frac{dI_{c}\left(t\right)}{dt}= & +\left(1-\epsilon_{H}\right)\gamma_{p}I_{p}-\gamma_{c}I_{c},\\ \frac{dH(t)}{dt}= & +\epsilon_{H}\gamma_{p}I_{p}-\gamma_{H}H,\\ \frac{dICU(t)}{dt}= & +\gamma_{H}\epsilon_{H2I}H-\gamma_{ICU}ICU,\\ \frac{dR(t)}{dt}= & +\gamma_{H}\left(1-\epsilon_{H2I}\right)H+\gamma_{ICU}\left(1-\epsilon_{x}\right)ICU+\gamma_{c}I_{c},\\ \frac{dX(t)}{dt}= & +\gamma_{ICU}\epsilon_{x}ICU,\\ \frac{dC(t)}{dt}= & +r_{d}^{p}\gamma_{p}I_{p}+\left(\frac{1-r_{d}^{p}}{1-\epsilon_{H}}\right)r_{d}^{c}\gamma_{c}I_{c}. \end{array} \end{array}$$ (1)

**Table 1:** Model parameters given with their descriptions, constrained ranges, and prior distributions.

Notation	Description	Constained range or definition	Prior distribution ‡
$R_{0}^{p}$	Basic reproduction number of the primarily infected population	0 − ∞	$R_{0}^{p}∼N\left(2.5,0.5\right)$
*r*_*c*_	Reduction in infectiousness due to being chronic	0%–100%	Fixed to a different value for each simulation.
$R_{0}^{c}$	Basic reproduction number of the chronically infected population	$R_{0}^{c}=R_{0}^{p}\left(1-r_{c}\right)$	Conditioned on $R_{0}^{p}$ and *r*_*c*_.
*r*_*L*_	Effect of lockdown in reducing infectiousness	0%–100%	*r*_*L*_ ∼ *β*(1, 1)
*m*_*L*_	Slope of reduction in infectiousness due to lockdown	0.5–1.5	*m*_*L*_ ∼ 0.5 + *β*(1, 1)
*s*_*L*_	Time lag of reduction in infectiousness due to lockdown	0 − ∞	*s*_*L*_ ∼ exp(1/5)
*r*_lock_(*t*)	Time dependent effect of the lockdown on the transmission rate	Given by Eq 2	Conditioned on *r*_*L*_, *r*_*c*_, *m*_*L*_, and *s*_*L*_.
1/*τ*	Duration of the latent period	0 − ∞	*τ* ∼ exp(1/2.5)
1/*γ*_*p*_	Duration of infection of *I*_*p*_	0 − ∞	*γ*_*p*_ ∼ exp(1/2.5)
1/*γ*_*c*_	Duration of infection of *I*_*c*_	0.01-20 days	Fixed to a different value for each simulation.
*β*_*p*_	Transmission rate of *I*_*p*_	Given by Eq 3	Conditioned on *r*_lock_(*t*), *R*_0_, and *γ*_*p*_.
*β*_*c*_	Transmission rate of *I*_*c*_	Given by Eq 4	Conditioned on *r*_lock_(*t*), *R*_0_, *γ*_*c*_, and *r*_*c*_.
1/*γ*_*H*_	Duration of hospital ward stay	0 − ∞	*γ*_*H*_ ∼ exp(1/12)
1/*γ*_*ICU*_	Duration of ICU stay	0 − ∞	*γ*_*ICU*_ ∼ exp(1/12)
*ϵ*_*H*_	Rate of direct H admission	0 − ∞	$\epsilon_{H}∼N\left(0.08,0.02\right)$
*ϵ*_*H*2*I*_	Transfer rate from H to ICU	0 − ∞	$\epsilon_{H2I}∼N\left(0.4,0.08\right)$
*ϵ*_*x*_	Death rate from ICU	0 − ∞	$\epsilon_{x}∼N\left(0.4,0.08\right)$
$r_{d}^{p}$	Diagnosis rate of *I*_*p*_	0 − ∞	$r_{d}^{p}∼N\left(0.2,0.03\right)$
$r_{d}^{c}$	Diagnosis rate of *I*_*c*_	0 − ∞	$r_{d}^{c}∼N\left(0.075,0.015\right)$
*R*_0_	Total basic reproduction number	$R_{0}=R_{0}^{p}+\left(1-\epsilon_{H}\right)R_{0}^{c}$	Conditioned on $R_{0}^{p}$, $R_{0}^{c}$ and *ϵ*_*H*_.
*E*(0)	Initial frequency of the exposed compartment	0%–100%	$r_{d}^{c}∼\beta \left(1,10^{3}\right)$
*S*(0)	Initial frequency of the susceptible compartment	1 − *E*(0)*	Conditioned on *E*(0)
*N*	Population size	−	Fixed specific to the country used for fitting.

* All other compartments (*I*_*p*_, *I*_*c*_, *H*, *ICU*, *R*, and *X*) are assumed to be zero at *t* = 0, and the first case is assumed to be observed at *t* = 1.

^‡^ N, *β*, exp denotes the Normal, Beta, and Exponential distributions respectively.

Time-dependent decrease in the transmission of SARS-CoV-2 due to lockdown measures is modeled by a sigmoid function [21], and denoted by *r*_*lock*_(*t*), such that

$$\begin{array}{cc} r_{lock}\left(t\right) & =r_{L}+\left(1-r_{L}\right)/[1+\exp(m_{L}\times \left(t-t_{L}-s_{L}\right))], \end{array}$$ (2)

where *r*_*L*_, *t*_*L*_, *m*_*L*_, and *s*_*L*_ denote the final effect of the lockdown, start date of the lockdown, slope of the decrease in transmissibility, and the time delay between implementation and effect of the lockdown, respectively. *r*_*lock*_(*t*) is used as a multiplicative factor in modeling the transmission rate in a time-dependent manner.

The reduced transmissibility of *I*_*c*_ is modeled via including a reduction coefficient *r*_*c*_ as a multiplicative factor to its transmission rate, representing the reduction in the infectiousness level of the primarily infected population when they move to the chronically infected phase. Introduction of *r*_*c*_ results in two different transmission rates *β*_*p*_ and *β*_*c*_ for *I*_*p*_ and *I*_*c*_ compartments, such that,

$$\begin{array}{cc} \beta_{p} & =r_{lock}\left(t\right)\times R_{0}^{p}\times \gamma_{p}, \end{array}$$ (3)

$$\begin{array}{cc} \beta_{c} & =r_{lock}\left(t\right)\times R_{0}^{c}\times \gamma_{c}, \end{array}$$ (4)

$$\begin{array}{cc} & =r_{lock}\left(t\right)\times R_{0}^{p}\left(1-r_{c}\right)\times \gamma_{c}, \end{array}$$ (5)

where $R_{0}^{p}$, $R_{0}^{c}$, 1/*γ*_*p*_, and 1/*γ*_*c*_ denotes the basic reproduction number of the primarily infected population, the basic reproduction number of the chronically infected population, duration of primarily infected phase, and the duration of chronically infected phase, respectively. We assume that individuals who develop symptoms do so only during the primarily infected phase, and therefore hospitalization is only possible before they transition to the chronically infected phase. We do not assume any a priori information regarding the testing policy, therefore a positive diagnosis is possible for both primarily and chronically infected individuals, and they contribute to the cumulative number of the positively diagnosed cases with the rates $r_{d}^{p}$ and $r_{d}^{c}$, respectively.

### Model fitting and parameter estimation

#### Model selection via goodness of fit until relaxation

We implemented two stages of model fitting. The first stage aims to compare the goodness of fits of three different classes of models, which are,

1. The model without prolonged viral shedding (model without the chronically infected (*I*_*c*_) compartment),
2. The model with prolonged viral shedding *without* prolonged infectiousness, where individuals in the *I*_*c*_ compartment are *not* infectious (model given by Eq set 1 for *r*_*c*_ = 100%, where *r*_*c*_ denotes the level of reduced infectiousness.),
3. The model with prolonged viral shedding and prolonged infectiousness, where individuals in the *I*_*c*_ compartment are infectious with different levels of infectiousness (given by Eq set 1 for 0% ≤ *r*_*c*_ < 100%).

The second model with *r*_*c*_ = 100% represents the scenario where the primarily infected individuals do not have prolonged infectiousness, but they still can be diagnosed during the chronic phase, meaning that their test results can still be positive although they are not infectious. Note that all models with prolonged viral shedding at different levels of infectiousness (0% ≤ *r*_*c*_ ≤ 100%) including the model without prolonged infectiousness at all (*r*_*c*_ = 100%) assume that the infected individuals are tested and positively diagnosed with a certain rate during this prolonged viral shedding window. This is not a common assumption in other modeling studies regarding COVID-19. Therefore, the first model without the chronically infected population is included in the comparison to represent the models which are conventionally used.

We then fit each class of model simultaneously to the data on the number of daily confirmed cases and the number of daily deaths. Due to the high spatial variation in transmission dynamics in countries such as Italy and the U.S., we used regional data within these two countries which have consistent spreading patterns. These regions include Lombardy, Piedmont, and Emilia-Romagna for Italy (data reported by the Civil Protection Department of the Ministry of Italy [22]), and the State of New York, the State of New Jersey, and the State of Louisiana for the U.S (data reported by the COVID Tracking Project [23]). Model fitting is done in a Bayesian framework using Stan [24]. The deviations between the model output and the data are assumed to follow a Negative Binomial distribution. Dispersion parameters of the Negative Binomial distributions are estimated separately for both the number of daily confirmed cases and the number of daily deaths during model fitting.

When fitting the models with prolonged viral shedding, we fixed the reduction in infectiousness parameter *r*_*c*_ to different values varying between 0% to 100%. Duration of infectiousness of the *I*_*c*_ compartment (1/*γ*_*c*_) is also fixed to different values varying from 0.01 to 20 days for all simulations. Other parameters are allowed to vary within their respective ranges, given in Table 1.

During model fitting, we use all the data points until the introduction of the easing on the lockdown measures (relaxation). We then calculate the Root Mean Squared Error (RMSE) between the median of the model estimates and the data points that are used for fitting to evaluate the goodness of the fit for the daily confirmed cases and the daily deaths for each class of model, where lower values of RMSE indicate a better fit.

RMSE values provide a good measure of fit by quantifying *how much* the median of the model estimate deviates from the data, and are useful to compare the goodness of fit of two different models. On the other hand, they do not incorporate the variance on the model estimates emerging from the probabilistic nature of the fitting procedure. To investigate *how often* one model performs better than another, we bootstrap estimates from both models within their 95% confidence intervals, and calculate the probability of one model having a greater error value than another model. Bootstrapping is performed via randomly subsampling simulated time series outputs with replacement for a given model. For each sample of a given model, we first normalize the RMSE values for the number of daily confirmed cases and the number of daily deaths via dividing them by the difference between the maximum and the minimum value of their respective data points. We then sum these normalized values up to calculate a combined measure of the goodness of fit, which we refer as the combined RMSE (CRMSE) value. We calculate the probability of one model having a greater CRMSE value than another model by comparing the CRMSE values of each bootstrapped sample for a given pair of models. This analysis is used to address two different questions. First, we investigate whether there is any advantage in including the chronically infected population in the model structure to achieve a better fit by calculating the probability of the model without the *I*_*c*_ compartment (model w/o *I*_*c*_) having a greater CRMSE value than the model with the *I*_*c*_ compartment for all levels of reduced infectiousness (0% ≤ *r*_*c*_ ≤ 100%), denoted by $P(CRMSE_{w/o\;I_{c}}>CRMSE_{r_{c}\leq 100\%})$. Second, we investigate whether there is a substantial difference between having prolonged infectiousness (*r*_*c*_ < 100%) versus being diagnosed without being infectious (*r*_*c*_ = 100%) during the prolonged viral shedding phase by calculating the probability of the model with *r*_*c*_ = 100% having a greater CRMSE value than the models with 0% ≤ *r*_*c*_ < 100%, denoted by *P*(*CRMSE*_*r*_*c*_ = 100%_ > *CRMSE*_*r*_*c*_ < 100%_). Both quantities are calculated for each duration of infectiousness (1/*γ*_*c*_ value) separately.

Due to the uncertainty of the quantitative effects of the easing on the lockdown measures (relaxation), data points after the relaxation are excluded from the goodness of fit calculations.

#### Model selection via predictive power after relaxation

The second stage of the model fitting aims to compare the predictive power of different models by incorporating the data after the introduction of relaxation. First, we use the data until relaxation provided by [25] for Switzerland, and fit the model simultaneously to four datasets: the number of daily confirmed cases, the number of daily deaths, the number of patients at the hospital ward at a given day, and the number of patients at the ICU at a given day. Using the parameters we obtained via fitting, we predict the number of daily confirmed cases in case of a gradual relaxation scenario for all models, using a range of *r*_*c*_ and *γ*_*c*_ values.

Relaxation is modeled as an increase in transmissibility, and characterized as a sigmoid function. It is similar to the time-dependent effect of the lockdown (*r*_*lock*_(*t*)) given by Eq 2, such that

$$\begin{array}{cc} r_{relax}\left(t\right) & =r_{L}+1/[1/\left(r_{\text{end}}-r_{L}\right)+\exp\left(-m_{R}\times \left(t-t_{R}-s_{R}\right)\right)], \end{array}$$ (6)

where *t*_*R*_, *m*_*R*_, *s*_*R*_, and *r*_end_ denote the start date of the relaxation (18^*th*^ of March for Switzerland, middle time point between the start of the first phase of relaxation on 27^*th*^ of April, and the start of the third—and the final—phase of relaxation on 8^*th*^ of June), slope of increase in transmissibility, the time delay until the effect of the relaxation takes place, and the final effect of the control measures still in place (wearing masks in public transit, practicing hand hygene, etc.), respectively. *r*_*relax*_(*t*) is used as a multiplicative factor in a similar fashion to *r*_*lock*_(*t*).

Since we aim to compare the predictive power of different models using the data after relaxation, parametrization of *r*_*relax*_(*t*) had to represent the quantitative impact of relaxation in Switzerland accurately, but also had to be independent of our model fitting procedure. Therefore, we parameterize *r*_*relax*_(*t*) using the effective reproductive number (*R*_*e*_(*t*)) estimates provided by the Swiss National COVID-19 Science Task Force [26], assuming that the normalized values of the effective reproductive number over time (*R*_*e*_(*t*)/*R*_*e*_(0)) provide a quantitative proxy for the change in behavior after the introduction of relaxation. We parameterize *r*_*relax*_(*t*) such that the numerical values for *m*_*R*_, *s*_*R*_, and *r*_end_ minimize the RMSE between *r*_*relax*_(*t*) and *R*_*e*_(*t*)/*R*_*e*_(0) for the time points (*t* values) after the introduction of relaxation. This parametrization is done separately for each model with different *r*_*c*_ and *γ*_*c*_ values, since their estimates for *r*_*L*_ will be different which is included in *r*_relax_(*t*) (Eq 6).

We quantify the predictive power of each model by calculating the RMSE values between the median of the model predictions and the daily confirmed cases data only for the time points after the introduction of relaxation. Similar to the first stage of model fitting, we calculate $P(RMSE_{w/o\;I_{c}}>RMSE_{r_{c}\leq 100\%})$ and $P(RMSE_{r_{c}=100\%}>RMSE_{r_{c}<100\%})$ values using the normalized RMSE results, but for the number of daily confirmed cases only. Because the uncertainty on the parameter estimates will propagate to the future predictions, prediction results will have wider confidence intervals than the fitting results. Therefore when calculating $P(RMSE_{w/o\;I_{c}}>RMSE_{r_{c}\leq 100\%})$ and $P(RMSE_{r_{c}=100\%}>RMSE_{r_{c}<100\%})$, we bootstrap prediction estimates within their 50% confidence intervals instead of 95% to identify the differences in model predictions in a more informative way (results with estimates bootstrapped within 95% confidence intervals are also provided in the Supporting Information). Model predictions for the other three data types (the number of daily deaths, the number of patients at the hospital ward at a given day, and the number of patients at the ICU at a given day) are excluded from the predictive power calculations, since the impact of relaxation manifests itself most directly in the number of daily confirmed cases data, whereas the other datasets are influenced by many other factors such as treatment success, demography of the patients, hospital capacity, etc. Such factors are likely to change over time and a re-fitting is required using the data points subsequent to the introduction of relaxation to estimate the related model parameters properly.

Only Swiss data is used to test the predictive power of different models because it is the only country to our knowledge where both the estimates on the effective reproductive number and the data on the hospitalized and the ICU patients are publicly available in high temporal resolution in addition to the number of daily confirmed cases and the number of daily deaths.

We implemented both stages of model fitting in a Bayesian framework using Stan [24]. Prior distributions of the parameters used during fitting are given in Table 1.

## Results

### Possibility of a chronically infected population

**Fig 2:**
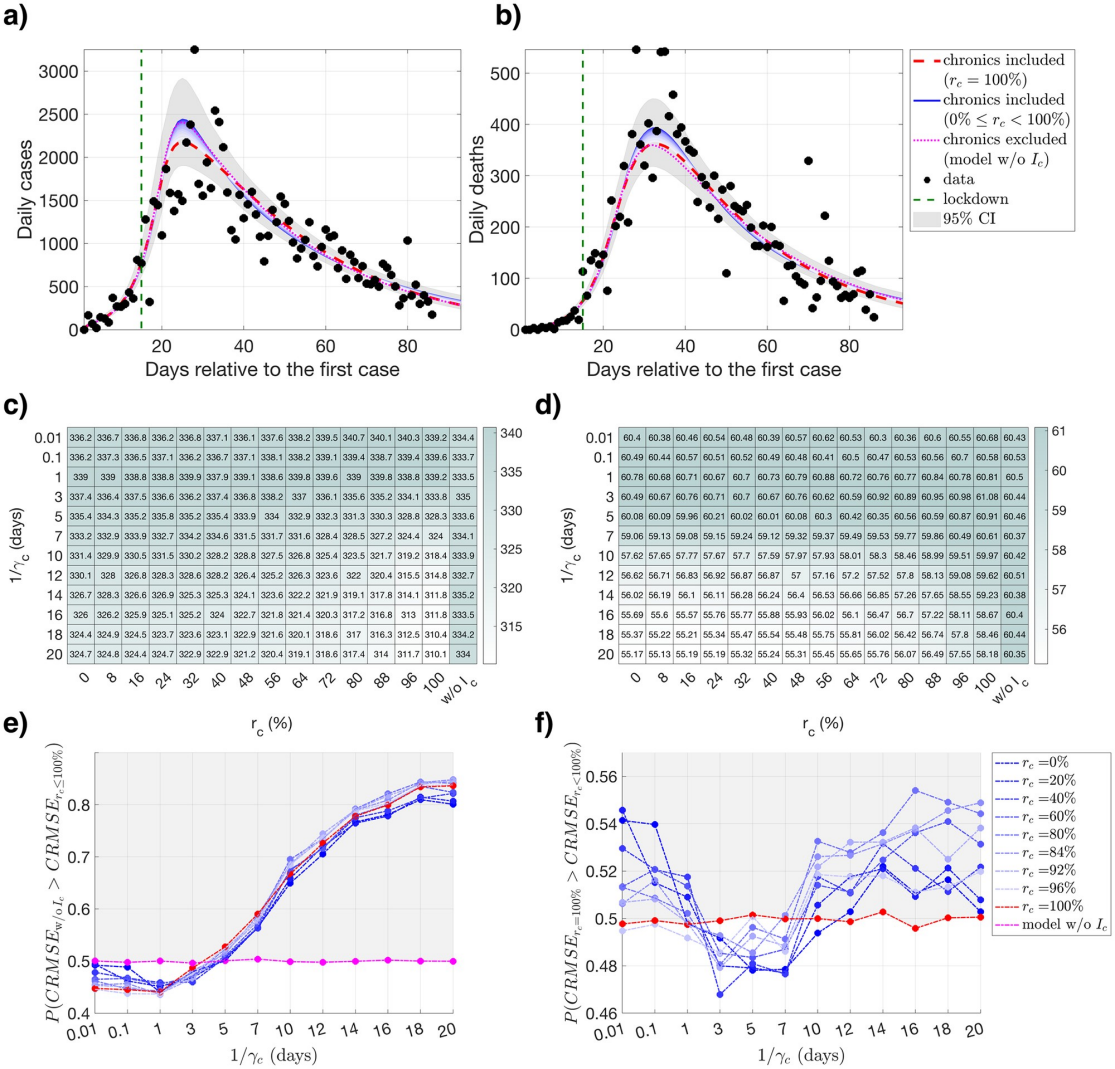
Fitting and Root Mean Squared Error (RMSE) results for Lombardy. Fitting and RMSE results for Lombardy, calculated using different levels and durations of infectiousness for the chronically infected population. Model outcomes (presented for 1/*γ*_*c*_ = 14 days) for the number of **a)** daily confimed cases and **b)** daily deaths using the data until the introduction of relaxation for model fitting, respectively. Darker shades of blue represent the fitting results with increased infectiousness of the chronically infected population, i.e., lower *r*_*c*_ values within the range 0 ≤ *r*_*c*_ < 100%. Fitting results for *r*_*c*_ = 100% are drawn in red, and the fitting results for the model without the *I*_*c*_ compartment (model w/o *I*_*c*_) are drawn in pink. Data points that are used for fitting are drawn in black. Gray areas around the model outcomes represent the union of the 95% confidence intervals calculated for all models. RMSE values **c)** for the number of daily confirmed cases and **d)** the number of daily deaths for a given *r*_*c*_ and *γ*_*c*_ value used for fitting, where model w/o *I*_*c*_ represents the results for the model without the *I*_*c*_ compartment. **e)** Probability of the model without the *I*_*c*_ compartment (model w/o *I*_*c*_) having a greater combined RMSE (CRMSE) value than the model with the *I*_*c*_ compartment for all levels of reduced infectiousness (*r*_*c*_ ≤ 100%) for different *r*_*c*_ and *γ*_*c*_ values. **f)** Probability of the model where individuals are being diagnosed without being infectious (*r*_*c*_ = 100%) having a greater combined RMSE (CRMSE) value than the model with individuals with a a prolonged infectiousness (*r*_*c*_ < 100%) for different *r*_*c*_ and *γ*_*c*_ values. Points in the gray areas represent the models that are providing a better fit more frequently than **e)** the model without the *I*_*c*_ compartment (model w/o *I*_*c*_) and **f)** the model with *r*_*c*_ = 100%.

We find that the model that describes the data the best is dependent on the combination of the level and duration of the prolonged infectiousness, and the optimal choice of the {*γ*_*c*_, *r*_*c*_} combination varies among different regions and different countries. For Lombardy, as the duration of infectiousness becomes longer, models including the chronically infected population outperform the model without the *I*_*c*_ compartment (model w/o *I*_*c*_) more often (Fig 2e), whereas the models with prolonged infectiousness (*r*_*c*_ < 100%) perform similarly to the model where the individuals can be diagnosed during the prolonged viral shedding window without being infectious (*r*_*c*_ = 100%) (Fig 2f). The absolute difference in RMSE values of the median of the model estimates differ by 30.7 daily confirmed cases (3.1% of the mean number of daily confirmed cases, S1 Table), and 6.17 daily deaths (3.4% of the mean number of daily deaths, S1 Table) the most when all *r*_*c*_ and *γ*_*c*_ values are considered (Fig 2c and 2d). The region of Emilia-Romagna presents a very similar behaviour to Lombardy (S1 Fig). In case of Piedmont and the state of New York, models with prolonged viral shedding (0% ≤ *r*_*c*_ ≤ 100%) outperform the model without the *I*_*c*_ compartment (model w/o *I*_*c*_) more often, and models with lower levels of infectiousness (higher *r*_*c*_ values) clearly provide a better fit than the models with higher level of infectiousness (S2 and S5 Figs). For the state of Louisiana, all models perform similarly (S3 Fig). For the state of New Jersey, RMSE values for the number of daily confirmed cases are sensitive to the particular combinations of *γ*_*c*_ and *r*_*c*_ values (S4 Fig). Model without the *I*_*c*_ compartment (model w/o *I*_*c*_) provides a better fit for very short and very long durations of prolonged infectiousness, and similar fits to the model with *r*_*c*_ = 100% for medium durations of prolonged infectiousness (S4 Fig). Maximum difference in the median RMSE values for the number of daily confirmed cases and the number of daily deaths for all combinations of *r*_*c*_ and *γ*_*c*_ values are provided in S1 Table, both in absolute values and relative to the mean of their corresponding data type. Parameter estimates with their corresponding means, standart deviations, and confidence intervals for all combinations of *r*_*c*_ and *γ*_*c*_ values are provided in the S2 Table.

### Impact of relaxation

**Fig 3:**
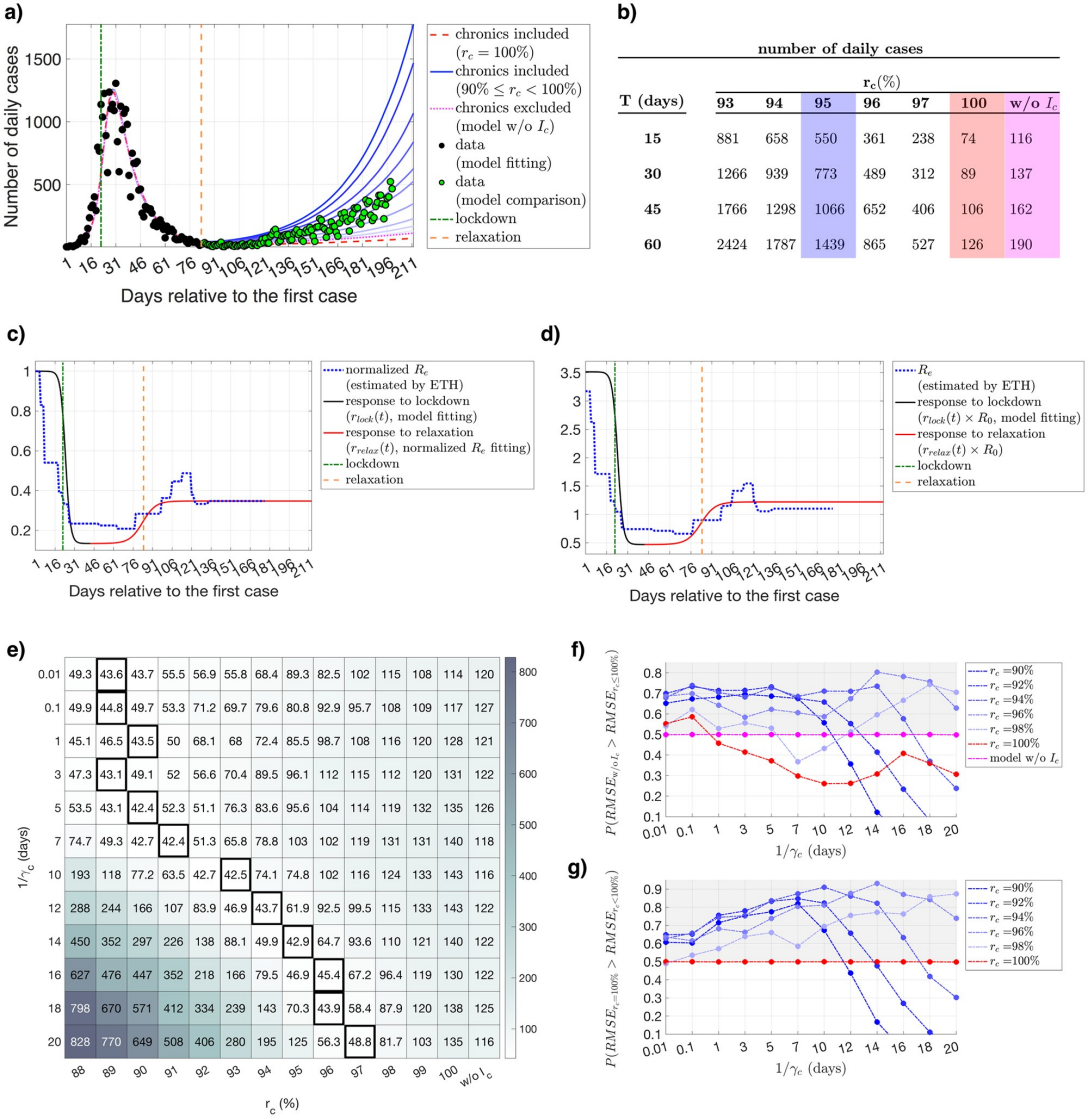
Fitting and prediction results for Switzerland. **a)** Fitting results and relaxation predictions for Switzerland for the number of daily confirmed cases, calculated using different levels of infectiousness for the chronically infected population, assuming a duration of prolonged infectiousness of 1/*γ*_*c*_ = 14 days. Time dependent effects of the lockdown (*r*_lock(*t*)_) and the relaxation (*r*_relax(*t*)_) are illustrated in **c)** and **d)**, respectively. Predictions drawn in darker shades of blue represent the fitting results with increased infectiousness of the chronically infected population, i.e., lower *r*_*c*_ values within the range 90 ≤ *r*_*c*_ < 100%. Fitting results for *r*_*c*_ = 100% are drawn in red, and the fitting results for the model without the *I*_*c*_ compartment (model w/o *I*_*c*_) are drawn in pink. Data points that are used for fitting are drawn in black, and the data points used for comparing the predictive power of different models are drawn in green. **e)** RMSE values calculated using the prediction results for the number of daily confirmed cases for a given *r*_*c*_ and *γ*_*c*_ value, where model w/o *I*_*c*_ represents the results for the model without the *I*_*c*_ compartment. Models with the best predictive power (smallest RMSE value) are indicated by the bold black boxes. **f)** Probability of the model without the *I*_*c*_ compartment (model w/o *I*_*c*_) having a greater RMSE value than the model with the *I*_*c*_ compartment for different levels of reduced infectiousness (90% ≤ *r*_*c*_ ≤ 100%) and *γ*_*c*_ values, calculated over the predicted data points. **g)** Probability of the model where individuals are being diagnosed without being infectious (*r*_*c*_ = 100%) having a greater RMSE value than the model with individuals with a a prolonged infectiousness (*r*_*c*_ < 100%) for different *r*_*c*_ and *γ*_*c*_ values, calculated over the predicted data points. Points in the gray areas represent the models that are providing a better fit more frequently than **f)** the model without the *I*_*c*_ compartment (model w/o *I*_*c*_) and **g)** the model with *r*_*c*_ = 100%. **b)** Prediction results of the first 5 models (*r*_*c*_ = {93%, 94%, 95%, 96%, 97%}) with the lowest RMSE value (best predictive power), model with *r*_*c*_ = 100%, and model without the *I*_*c*_ compartment (model w/o *I*_*c*_) for + *T* days into the future from the last data point observed, where 1/*γ*_*c*_ = 14 days. Predictions for the model with the best predictive power (*r*_*c*_ = 95%), for the model with *r*_*c*_ = 100%, and the model without the *I*_*c*_ compartment (model w/o *I*_*c*_) are highlighted in blue, red, and pink, respectively.

Data for Switzerland after the introduction of relaxation is used to test the predictive power of different models. As a demonstrative example, effect of the lockdown and the relaxation on infectiousness (*r*_lock_(*t*) and *r*_relax_(*t*)) are provided in Fig 3c and 3d for 1/*γ*_*c*_ = 14 days.

We observe that all models provide almost identical fits for the data prior to the introduction of relaxation (S6 Fig), but they substantially differ in their predictions regarding after relaxation even for small differences in the infectiousness levels (*r*_*c*_ values) for the chronically infected population (Fig 3a and 3b, demonstrative example for 1/*γ*_*c*_ = 14 days). As the predicted point moves further in time, the quantitative difference between the predictions of different models deviate from each other even more. For 60 days after the last data point observed, the model with the best predictive power (model with *r*_*c*_ = 95%) predicts a median of 1439 daily confirmed cases, whereas model with *r*_*c*_ = 100% predicts a median of 126, and model without the *I*_*c*_ compartment (model w/o *I*_*c*_) predicts a median of 190 daily confirmed cases (Fig 3b, *T* = 60), indicating a discrepancy by one order of magnitude. Similar to the results provided for the first stage of fitting, we find that it is more advantageous to use a model including the chronically infected population with low levels of infectiousness (Fig 3f and 3g for estimates bootstrapped within 50%, and S7 Fig for estimates bootstrapped within 95% confidence intervals). The model providing the lowest RMSE value between the predictions and the data is always a model with prolonged infectiousness for a wide range of *γ*_*c*_ values (Fig 3e). However, note that these results are valid under our particular choice of parametrization of the relaxation dynamics, and the resulting relative change in the transmissibility during the relaxation period.

The fact that observed data can be explained equally well by various combinations of *r*_*c*_ and *γ*_*c*_ values is partially due to the flexibility of the fitting procedure, which allows other parameters to be adjusted for a given {*r*_*c*_, *γ*_*c*_} pair. Most parameters are free to vary, but their prior distributions are informed such that the hyperparameters (parameters of the prior distributions) align with the reported values in the literature (Table 1). As an example, both the incubation period (1/*τ*) and the duration of infectiousness of the primarily infected population (1/*γ*_*p*_) have the mean of 2.5 days, resulting in a generation time distribution with a mean of 5 days, in agreement with the reported values in the literature for COVID-19 (see Introduction). Similarly, the basic reproduction number of the primarily infected population $R_{0}^{p}$ is normally distributed with a mean of 2.5, which is the average value reported for basic reproduction number of COVID-19 in many countries [10, 27]. Mean values of the prior distributions of the parameters related to hospitalization (*γ*_*H*_, *γ*_*ICU*_, *ϵ*_*H*_, *ϵ*_*H*2*I*_, and *ϵ*_*x*_) are adopted from Ferguson *et al*. [28] and Verity *et al*. [29], and given a variance such that they can be adjusted specifically for each country during the fitting procedure.

**Fig 4:**
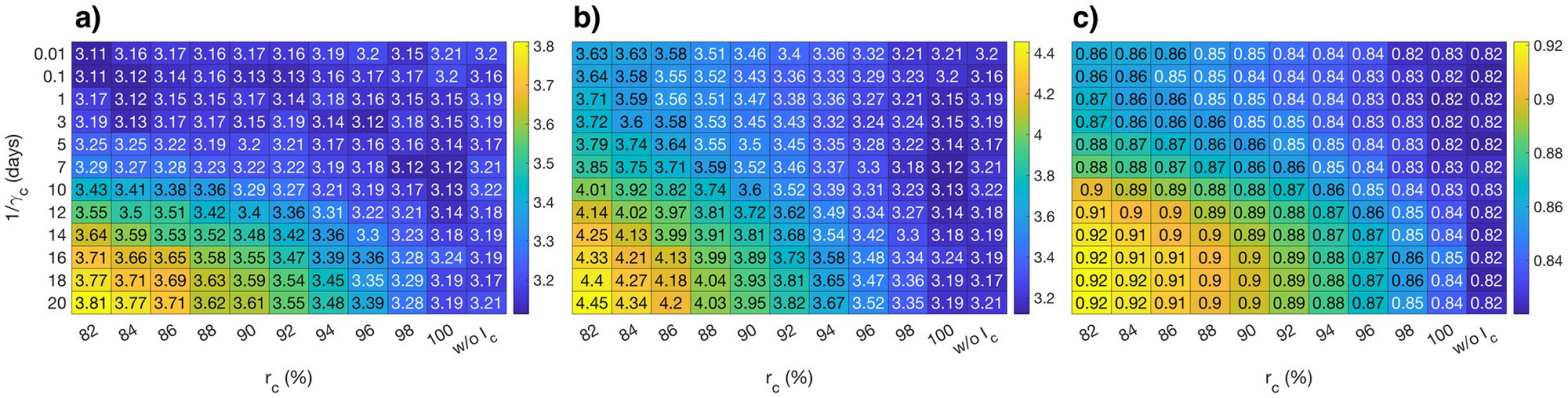
Median of the posterior distributions of parameters. Median values of the posterior distributions of the **a)** basic reproduction number of the primarily infected population ($R_{0}^{p}$), **b)** total basic reproduction number ($R_{0}=R_{0}^{p}+\left(1-\epsilon_{H}\right)R_{0}^{c}$), and **c)** the final reduction in infectiousness due to lockdown (1 − *r*_*L*_), for a given {*r*_*c*_, *γ*_*c*_} pair.

The median of the posterior distributions for $R_{0}^{p}$, *R*_0_, and *r*_*L*_ provide a good example to demonstrate the flexibility of the fitting procedure (Fig 4). As expected, the basic reproduction number of the primarily infected population ($R_{0}^{p}$) (Fig 4a), the total basic reproduction number ($R_{0}=R_{0}^{p}+\left(1-\epsilon_{H}\right)R_{0}^{c}$) (Fig 4b), and the final reduction in infectiousness due to lockdown (1 − *r*_*L*_) (Fig 4c) are estimated to be lower for a given duration of infectiousness (1/*γ*_*c*_) as the infectiousness of the chronically infected population decreases (as *r*_*c*_ increases) to explain the observed data.

Parameter estimates for the Swiss data with their corresponding means, standart deviations, and confidence intervals for all combinations of *r*_*c*_ and *γ*_*c*_ values are provided in the S2 Table.

## Discussion

The model presented in this work explores the plausibility of an epidemiological model with a prolonged viral shedding window for the COVID-19 pandemic, and investigates both its impact and predictive capabilities on the outcomes of a gradual easing on the lockdown measures (relaxation) given different assumptions on the infectiousness level and duration of a chronically infected population.

Our results show that including a chronically infected population, i.e., individuals that are less infectious but infectious for a longer duration, is not a possibility that can be easily rejected from an epidemiological perspective. This conclusion is based on two main results. First, neither the presence nor the absence of chronic transmission is identifiable from population-level data. The data that has been observed until relaxation can be explained equally well by the model with prolonged viral shedding for a variety of different levels and durations of prolonged infectiousness as by the model without prolonged viral shedding. Although this is partially due to the flexibility of the fitting procedure, the choice of hyperparameters (parameters of the prior distributions) indicates that all fits for a given infectiousness value are possible for a set of reasonable model parameters, and therefore as favorable as the conventional models from a modeling perspective.

Second, even if the presence of a chronically infected population cannot be proven, its introduction to the model structure has a considerable impact on the relaxation outcomes. In case of a gradual easing on the lockdown measures, the predictions of the model including the chronically infected population vary considerably from those obtained under a model in which prolonged infectiousness is not taken into account. Although the level of infectiousness might be low, its impact during the prolonged viral shedding window is significant in terms of predicting the outcomes of a gradual relaxation, indicating that even small differences in prolonged infectiousness levels might change the course of an epidemic when they are present for a certain duration. This is especially important for the healthcare systems if projections such as the occupancy of the hospital ward or the ICU are calculated using similar dynamical models.

The fact that observed data can also be explained with a model including prolonged viral shedding raises certain questions about the interpretation of the epidemic curve, acquired immunity, and the current testing policies. Assuming a relatively short generation time for a model that does not consider a prolonged viral shedding window results in more optimistic projections about epidemic control, as clearly demonstrated in Fig 3. Based on this, countries that were very successful in their initial control measures and therefore experienced a very steep decline in the number of daily confirmed cases might choose to ease the control measures too soon. We still lack a full understanding of the viral shedding window of COVID-19, and therefore might have a biased opinion on the number of infectious individuals in the community. This once again emphasizes the infectiousness of COVID-19 and the significance of frequent testing although the number of cases are in decline.

Using simplified compartmental models such as the one in this study has certain limitations. First, it does not consider the stochastic effects that the system is subject to, which become more important as the number of infecteds decrease in the community. Second, it assumes a well-mixed population, and does not consider the contact structure and the demographic information which are both relevant to the disease spread. Nevertheless, we believe that these two drawbacks of our modeling approach influence all models with and without the prolonged viral shedding to a similar degree, if not penalizing the models with prolonged viral shedding for producing more pessimistic projections since the number of infecteds will be higher in frequency relative to the model without the chronically infected compartment. Additionally, the standard SEIR model assumes constant rates of transition between the exposed, infectious, and recovered classes, leading to waiting times that individuals spend in these states being exponentially distributed [30], resulting in an exponential distribution on the generation time as well. Although mathematically convenient, this assumption is shown to be epidemiologically unrealistic, and less dispersed distributions such as gamma distribution should be used instead [31, 32].

Compartmental model structures are based on the underlying epidemiological and demographic interactions of a particular disease. Given that there are many choices for these interactions, the number of possible combinations are enormous [33, 34]. Our choice of including a chronically infected comparment in the model structure was inspired by the evidence indicating an uncertainity regarding the generation time distributions, but this approach is only one way to extend the basic SEIR model for the COVID-19 pandemic. There are still several open questions regarding the transmission dynamics of COVID-19, meaning that there are many other alternatives of modification a modeler could consider depending on the research question in hand. These alternatives are also potential candidates which would describe the data equally well, and offer reasonable predictions.

One methodological limitation of pure model fitting is the parameter identification problem, especially in the early stages of an epidemic [35]. As clearly demonstrated in Fig 4, models with different assumptions on the duration and the level of prolonged infectiousness lead to equally good descriptions of the observed data by adjusting the parameter values accordingly. Therefore, even if a prolonged viral shedding window exists for COVID-19, it would not be possible to quantify the precise level or the duration of prolonged infectiousness by using model fitting purely. Ultimately, these quantities should be measured or estimated from the relevant type of data.

Another potential limitation is the dependency of the goodness of fit of a given model on the quantification of the impact of relaxation, which inevitably affects the model selection procedure. Although we do not perform any fitting on the data belonging to the relaxation phase, we indirectly inform our predictions by shaping the change in transmissibility (*β*) via using the normalized values of the *R*_*e*_ estimates provided by the Swiss National COVID-19 Science Task Force, which in turn are calculated using the data on the number of daily cases. Different assumptions on the change of transmissibility during relaxation might alter the infectiousness level that is optimal in predicting the relaxation outcomes. With that being said, our results suggest that the model without the chronically infected compartment heavily underpredicts the case numbers, as clearly seen in Fig 3. Although it is still debated whether the patients who recover from COVID-19 and test positive for the virus after their recovery are still infectious or not, it is clear that these positive test results contribute to the data on the number of daily confirmed cases. However, current modeling studies regarding COVID-19 neglect this fact and assume that the probability of detecting an infection decreases strongly after the mean generation time. Our results show that this assumption might lead to an underestimation of both the reproduction number and the effect of the lockdown (Fig 4), leading to a potential underprediction for the relaxation outcomes.

In conclusion, it is not possible to either prove or disprove the existence of a compartment of chronically infected individuals purely by modeling based on epidemiological data. Our results only provide tentative evidence to consider a chronically infected population as an alternative modeling approach in addressing the knowledge gap on the transmission dynamics of COVID-19. Such an hypothesis must be tested by incorporating data regarding the timing of transmission events, contact histories, and corresponding test results. Furthermore, more clinical and virological diagnostic studies are necessary to establish the biological links between viral load, active viral replication at different sites of the body, severity of symptoms, and a positive test result to infer the infectiousness of an individual over time. Including a chronically infected population in our model was motivated by the evidence reported for prolonged viral shedding in the literature [14–20], and attempted to test whether this is also a plausible descriptive and predictive modeling approach. Given that different assumptions on the infectiousness duration and level during a prolonged viral shedding window can result in similar descriptions of the observed data prior to the introduction of relaxation, and large differences of epidemic projections after relaxation, it is important to consider a chronically infected population from a modeling perspective when national policies are being imposed.

## Supporting information

S1 Fig Fitting and Root Mean Squared Error (RMSE) results for Emilia-Romagna. Fitting and RMSE results for Emilia-Romagna, calculated using different levels and durations of infectiousness for the chronically infected population. Model outcomes (presented only for 1/*γ*_*c*_ = 14 days) for the number of **a)** daily confimed cases and **b)** daily deaths using the data until the introduction of relaxation for model fitting, respectively. Darker shades of blue represent the fitting results with increased infectiousness of the chronically infected population, i.e., lower *r*_*c*_ values within the range 0 ≤ *r*_*c*_ < 100%. Fitting results for *r*_*c*_ = 100% are drawn in red, and the fitting results for the model without the *I*_*c*_ compartment (model w/o *I*_*c*_) are drawn in pink. Data points that are used for fitting are drawn in black. Gray areas around the model outcomes represent the union of the 95% confidence intervals calculated for all models. RMSE values **c)** for the number of daily confirmed cases and **d)** the number of daily deaths for a given *r*_*c*_ and *γ*_*c*_ value used for fitting, where model w/o *I*_*c*_ represents the results for the model without the *I*_*c*_ compartment. **e)** Probability of the model without the *I*_*c*_ compartment (model w/o *I*_*c*_) having a greater combined RMSE (CRMSE) value than the model with the *I*_*c*_ compartment for all levels of reduced infectiousness (*r*_*c*_ ≤ 100%) for different *r*_*c*_ and *γ*_*c*_ values. **f)** Probability of the model where individuals are being diagnosed without being infectious (*r*_*c*_ = 100%) having a greater combined RMSE (CRMSE) value than the model with individuals with a a prolonged infectiousness (*r*_*c*_ < 100%) for different *r*_*c*_ and *γ*_*c*_ values. Points in the gray areas represent the models that are providing a better fit more frequently than **e)** the model without the *I*_*c*_ compartment (model w/o *I*_*c*_) and **f)** the model with *r*_*c*_ = 100%.
(TIFF)

S2 Fig Fitting and Root Mean Squared Error (RMSE) results for Piedmont. Fitting and RMSE results for Piedmont, calculated using different levels and durations of infectiousness for the chronically infected population. Model outcomes (presented only for 1/*γ*_*c*_ = 14 days) for the number of **a)** daily confimed cases and **b)** daily deaths using the data until the introduction of relaxation for model fitting, respectively. Darker shades of blue represent the fitting results with increased infectiousness of the chronically infected population, i.e., lower *r*_*c*_ values within the range 0 ≤ *r*_*c*_ < 100%. Fitting results for *r*_*c*_ = 100% are drawn in red, and the fitting results for the model without the *I*_*c*_ compartment (model w/o *I*_*c*_) are drawn in pink. Data points that are used for fitting are drawn in black. Gray areas around the model outcomes represent the union of the 95% confidence intervals calculated for all models. RMSE values **c)** for the number of daily confirmed cases and **d)** the number of daily deaths for a given *r*_*c*_ and *γ*_*c*_ value used for fitting, where model w/o *I*_*c*_ represents the results for the model without the *I*_*c*_ compartment. **e)** Probability of the model without the *I*_*c*_ compartment (model w/o *I*_*c*_) having a greater combined RMSE (CRMSE) value than the model with the *I*_*c*_ compartment for all levels of reduced infectiousness (*r*_*c*_ ≤ 100%) for different *r*_*c*_ and *γ*_*c*_ values. **f)** Probability of the model where individuals are being diagnosed without being infectious (*r*_*c*_ = 100%) having a greater combined RMSE (CRMSE) value than the model with individuals with a a prolonged infectiousness (*r*_*c*_ < 100%) for different *r*_*c*_ and *γ*_*c*_ values. Points in the gray areas represent the models that are providing a better fit more frequently than **e)** the model without the *I*_*c*_ compartment (model w/o *I*_*c*_) and **f)** the model with *r*_*c*_ = 100%.
(TIFF)

S3 Fig Fitting and Root Mean Squared Error (RMSE) results for the State of Louisiana. Fitting and RMSE results for the State of Louisiana, calculated using different levels and durations of infectiousness for the chronically infected population. Model outcomes (presented only for 1/*γ*_*c*_ = 14 days) for the number of **a)** daily confimed cases and **b)** daily deaths using the data until the introduction of relaxation for model fitting, respectively. Darker shades of blue represent the fitting results with increased infectiousness of the chronically infected population, i.e., lower *r*_*c*_ values within the range 0 ≤ *r*_*c*_ < 100%. Fitting results for *r*_*c*_ = 100% are drawn in red, and the fitting results for the model without the *I*_*c*_ compartment (model w/o *I*_*c*_) are drawn in pink. Data points that are used for fitting are drawn in black. Gray areas around the model outcomes represent the union of the 95% confidence intervals calculated for all models. RMSE values **c)** for the number of daily confirmed cases and **d)** the number of daily deaths for a given *r*_*c*_ and *γ*_*c*_ value used for fitting, where model w/o *I*_*c*_ represents the results for the model without the *I*_*c*_ compartment. **e)** Probability of the model without the *I*_*c*_ compartment (model w/o *I*_*c*_) having a greater combined RMSE (CRMSE) value than the model with the *I*_*c*_ compartment for all levels of reduced infectiousness (*r*_*c*_ ≤ 100%) for different *r*_*c*_ and *γ*_*c*_ values. **f)** Probability of the model where individuals are being diagnosed without being infectious (*r*_*c*_ = 100%) having a greater combined RMSE (CRMSE) value than the model with individuals with a a prolonged infectiousness (*r*_*c*_ < 100%) for different *r*_*c*_ and *γ*_*c*_ values. Points in the gray areas represent the models that are providing a better fit more frequently than **e)** the model without the *I*_*c*_ compartment (model w/o *I*_*c*_) and **f)** the model with *r*_*c*_ = 100%.
(TIFF)

S4 Fig Fitting and Root Mean Squared Error (RMSE) results for the State of New Jersey. Fitting and RMSE results for the State of New Jersey, calculated using different levels and durations of infectiousness for the chronically infected population. Model outcomes (presented only for 1/*γ*_*c*_ = 14 days) for the number of **a)** daily confimed cases and **b)** daily deaths using the data until the introduction of relaxation for model fitting, respectively. Darker shades of blue represent the fitting results with increased infectiousness of the chronically infected population, i.e., lower *r*_*c*_ values within the range 0 ≤ *r*_*c*_ < 100%. Fitting results for *r*_*c*_ = 100% are drawn in red, and the fitting results for the model without the *I*_*c*_ compartment (model w/o *I*_*c*_) are drawn in pink. Data points that are used for fitting are drawn in black. Gray areas around the model outcomes represent the union of the 95% confidence intervals calculated for all models. RMSE values **c)** for the number of daily confirmed cases and **d)** the number of daily deaths for a given *r*_*c*_ and *γ*_*c*_ value used for fitting, where model w/o *I*_*c*_ represents the results for the model without the *I*_*c*_ compartment. **e)** Probability of the model without the *I*_*c*_ compartment (model w/o *I*_*c*_) having a greater combined RMSE (CRMSE) value than the model with the *I*_*c*_ compartment for all levels of reduced infectiousness (*r*_*c*_ ≤ 100%) for different *r*_*c*_ and *γ*_*c*_ values. **f)** Probability of the model where individuals are being diagnosed without being infectious (*r*_*c*_ = 100%) having a greater combined RMSE (CRMSE) value than the model with individuals with a a prolonged infectiousness (*r*_*c*_ < 100%) for different *r*_*c*_ and *γ*_*c*_ values. Points in the gray areas represent the models that are providing a better fit more frequently than **e)** the model without the *I*_*c*_ compartment (model w/o *I*_*c*_) and **f)** the model with *r*_*c*_ = 100%.
(TIFF)

S5 Fig Fitting and Root Mean Squared Error (RMSE) results for the State of New York. Fitting and RMSE results for the State of New York, calculated using different levels and durations of infectiousness for the chronically infected population. Model outcomes (presented only for 1/*γ*_*c*_ = 14 days) for the number of **a)** daily confimed cases and **b)** daily deaths using the data until the introduction of relaxation for model fitting, respectively. Darker shades of blue represent the fitting results with increased infectiousness of the chronically infected population, i.e., lower *r*_*c*_ values within the range 0 ≤ *r*_*c*_ < 100%. Fitting results for *r*_*c*_ = 100% are drawn in red, and the fitting results for the model without the *I*_*c*_ compartment (model w/o *I*_*c*_) are drawn in pink. Data points that are used for fitting are drawn in black. Gray areas around the model outcomes represent the union of the 95% confidence intervals calculated for all models. RMSE values **c)** for the number of daily confirmed cases and **d)** the number of daily deaths for a given *r*_*c*_ and *γ*_*c*_ value used for fitting, where model w/o *I*_*c*_ represents the results for the model without the *I*_*c*_ compartment. **e)** Probability of the model without the *I*_*c*_ compartment (model w/o *I*_*c*_) having a greater combined RMSE (CRMSE) value than the model with the *I*_*c*_ compartment for all levels of reduced infectiousness (*r*_*c*_ ≤ 100%) for different *r*_*c*_ and *γ*_*c*_ values. **f)** Probability of the model where individuals are being diagnosed without being infectious (*r*_*c*_ = 100%) having a greater combined RMSE (CRMSE) value than the model with individuals with a a prolonged infectiousness (*r*_*c*_ < 100%) for different *r*_*c*_ and *γ*_*c*_ values. Points in the gray areas represent the models that are providing a better fit more frequently than **e)** the model without the *I*_*c*_ compartment (model w/o *I*_*c*_) and **f)** the model with *r*_*c*_ = 100%.
(TIFF)

S6 Fig Root Mean Squared Error (RMSE) results of model fitting prior to the introduction of relaxation for Switzerland. RMSE results of model fitting prior to the introduction of relaxation for Switzerland, calculated using different levels and durations of infectiousness for the chronically infected population.
(TIFF)

S7 Fig Comparison of RMSE values for Switzerland for 95% confidence interval Bootstrapping. **a)** Probability of the model without the *I*_*c*_ compartment (model w/o *I*_*c*_) having a greater RMSE value than the model with the *I*_*c*_ compartment for different levels of reduced infectiousness (90%≤*r*_*c*_ ≤ 100%) and *γ*_*c*_ values, calculated over the predicted data points. **b)** Probability of the model where individuals are being diagnosed without being infectious (*r*_*c*_ = 100%) having a greater RMSE value than the model with individuals with a a prolonged infectiousness (*r*_*c*_ < 100%) for different *r*_*c*_ and *γ*_*c*_ values, calculated over the predicted data points. Points in the gray areas represent the models that are providing a better fit more frequently than **a)** the model without the *I*_*c*_ compartment (model w/o *I*_*c*_) and **b)** the model with *r*_*c*_ = 100%.
(TIFF)

S1 Table Maximum differences in the median RMSE values. Maximum difference in the median RMSE values for the number of daily confirmed cases and the number of daily deaths for all combinations of *r*_*c*_ and *γ*_*c*_ values for Lombardy, Emilia-Romagna, Piedmont, State of Louisiana, State of New Jersey, and the State of New York. RMSE values are provided in both in absolute values and relative to the mean of their corresponding data type.
(XLSX)

S2 Table Parameter estimates with their corresponding means, standart deviations, and confidence intervals. Parameter estimates with their corresponding means, standart deviations, and confidence intervals for all combinations of *r*_*c*_ and *γ*_*c*_ values for Lombardy, Emilia-Romagna, Piedmont, State of Louisiana, State of New Jersey, the State of New York, and Switzerland.
(XLSX)
