# Supplementary figures and images for: Assessing the potential impact of transmission during prolonged viral shedding on the effect of lockdown relaxation on COVID-19
Source: PLoS Comput Biol. 2021 Jan 29;17(1):e1008609. doi: 10.1371/journal.pcbi.1008609 (PMC7875355; doi:10.1371/journal.pcbi.1008609)

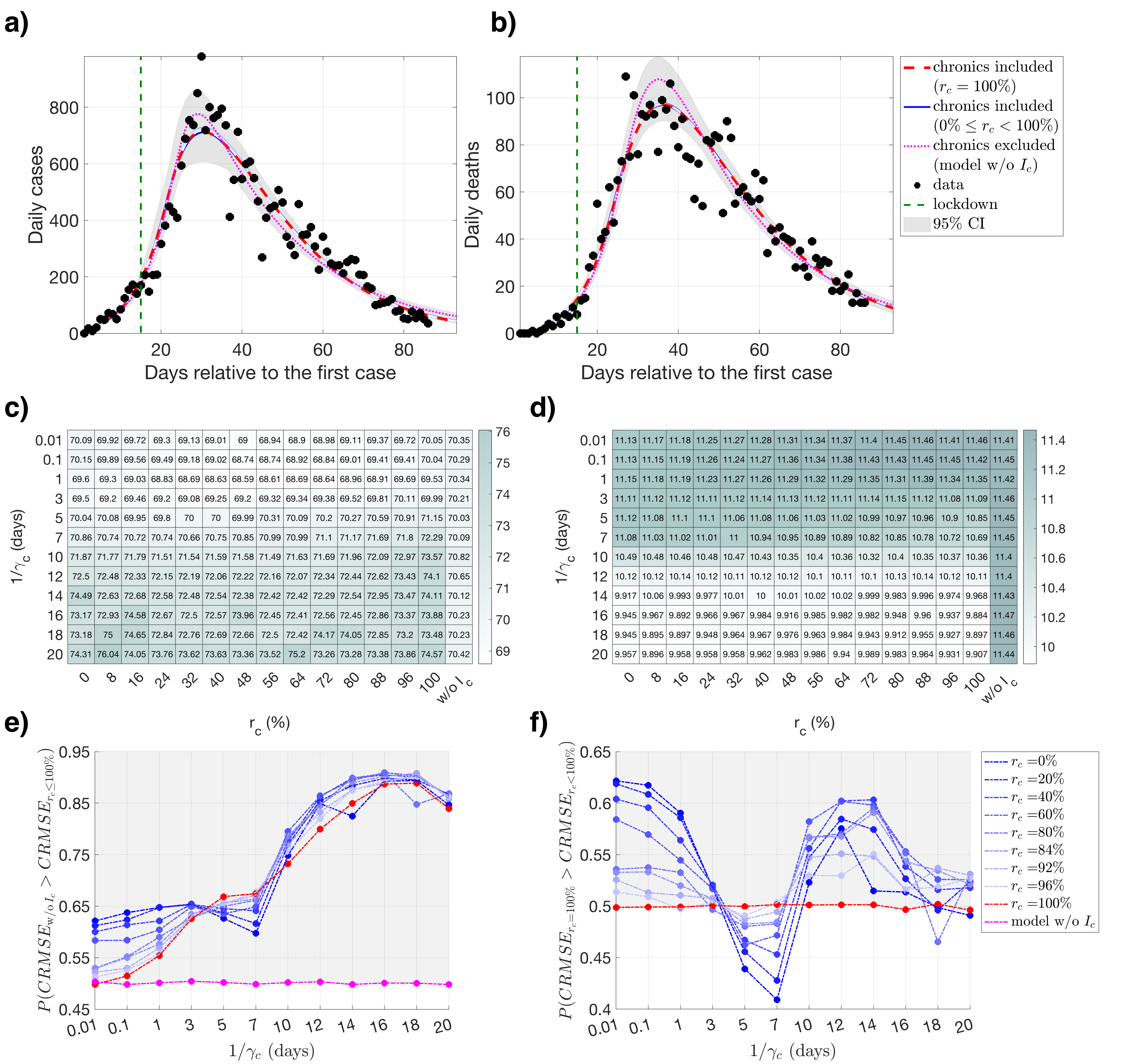

Supplement: S1 Fig — Fitting and RMSE results for Emilia-Romagna, calculated using different levels and durations of infectiousness for the chronically infected population. Model outcomes (presented only for 1/γc = 14 days) for the number of a) daily confimed cases and b) daily deaths using the data until the introduction of relaxation for model fitting, respectively. Darker shades of blue represent the fitting results with increased infectiousness of the chronically infected population, i.e., lower rc values within the range 0 ≤ rc < 100%. Fitting results for rc = 100% are drawn in red, and the fitting results for the model without the Ic compartment (model w/o Ic) are drawn in pink. Data points that are used for fitting are drawn in black. Gray areas around the model outcomes represent the union of the 95% confidence intervals calculated for all models. RMSE values c) for the number of daily confirmed cases and d) the number of daily deaths for a given rc and γc value used for fitting, where model w/o Ic represents the results for the model without the Ic compartment. e) Probability of the model without the Ic compartment (model w/o Ic) having a greater combined RMSE (CRMSE) value than the model with the Ic compartment for all levels of reduced infectiousness (rc ≤ 100%) for different rc and γc values. f) Probability of the model where individuals are being diagnosed without being infectious (rc = 100%) having a greater combined RMSE (CRMSE) value than the model with individuals with a a prolonged infectiousness (rc < 100%) for different rc and γc values. Points in the gray areas represent the models that are providing a better fit more frequently than e) the model without the Ic compartment (model w/o Ic) and f) the model with rc = 100%. (TIFF) [file pcbi.1008609.s001.tiff]

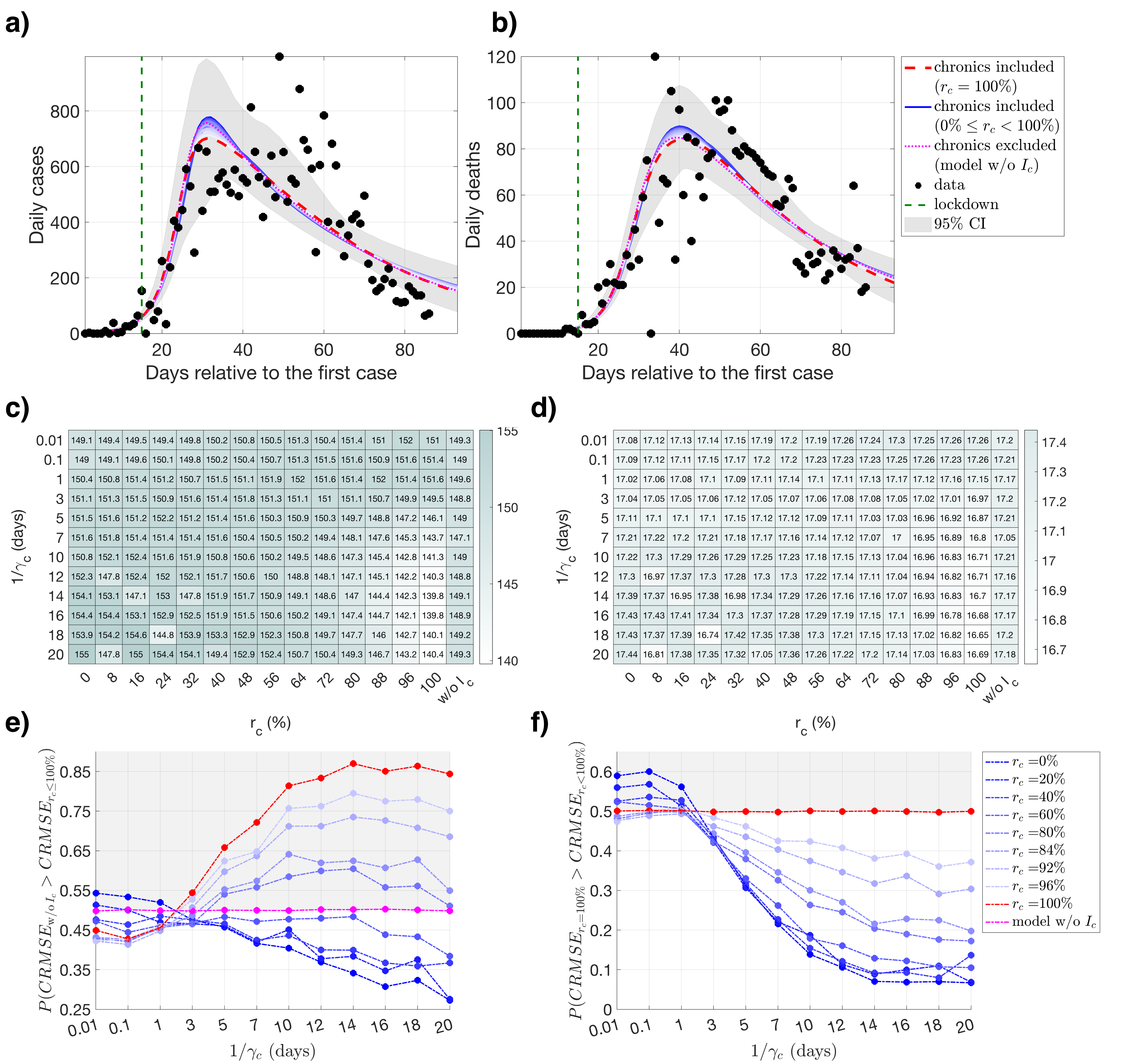

Supplement: S2 Fig — Fitting and RMSE results for Piedmont, calculated using different levels and durations of infectiousness for the chronically infected population. Model outcomes (presented only for 1/γc = 14 days) for the number of a) daily confimed cases and b) daily deaths using the data until the introduction of relaxation for model fitting, respectively. Darker shades of blue represent the fitting results with increased infectiousness of the chronically infected population, i.e., lower rc values within the range 0 ≤ rc < 100%. Fitting results for rc = 100% are drawn in red, and the fitting results for the model without the Ic compartment (model w/o Ic) are drawn in pink. Data points that are used for fitting are drawn in black. Gray areas around the model outcomes represent the union of the 95% confidence intervals calculated for all models. RMSE values c) for the number of daily confirmed cases and d) the number of daily deaths for a given rc and γc value used for fitting, where model w/o Ic represents the results for the model without the Ic compartment. e) Probability of the model without the Ic compartment (model w/o Ic) having a greater combined RMSE (CRMSE) value than the model with the Ic compartment for all levels of reduced infectiousness (rc ≤ 100%) for different rc and γc values. f) Probability of the model where individuals are being diagnosed without being infectious (rc = 100%) having a greater combined RMSE (CRMSE) value than the model with individuals with a a prolonged infectiousness (rc < 100%) for different rc and γc values. Points in the gray areas represent the models that are providing a better fit more frequently than e) the model without the Ic compartment (model w/o Ic) and f) the model with rc = 100%. (TIFF) [file pcbi.1008609.s002.tiff]

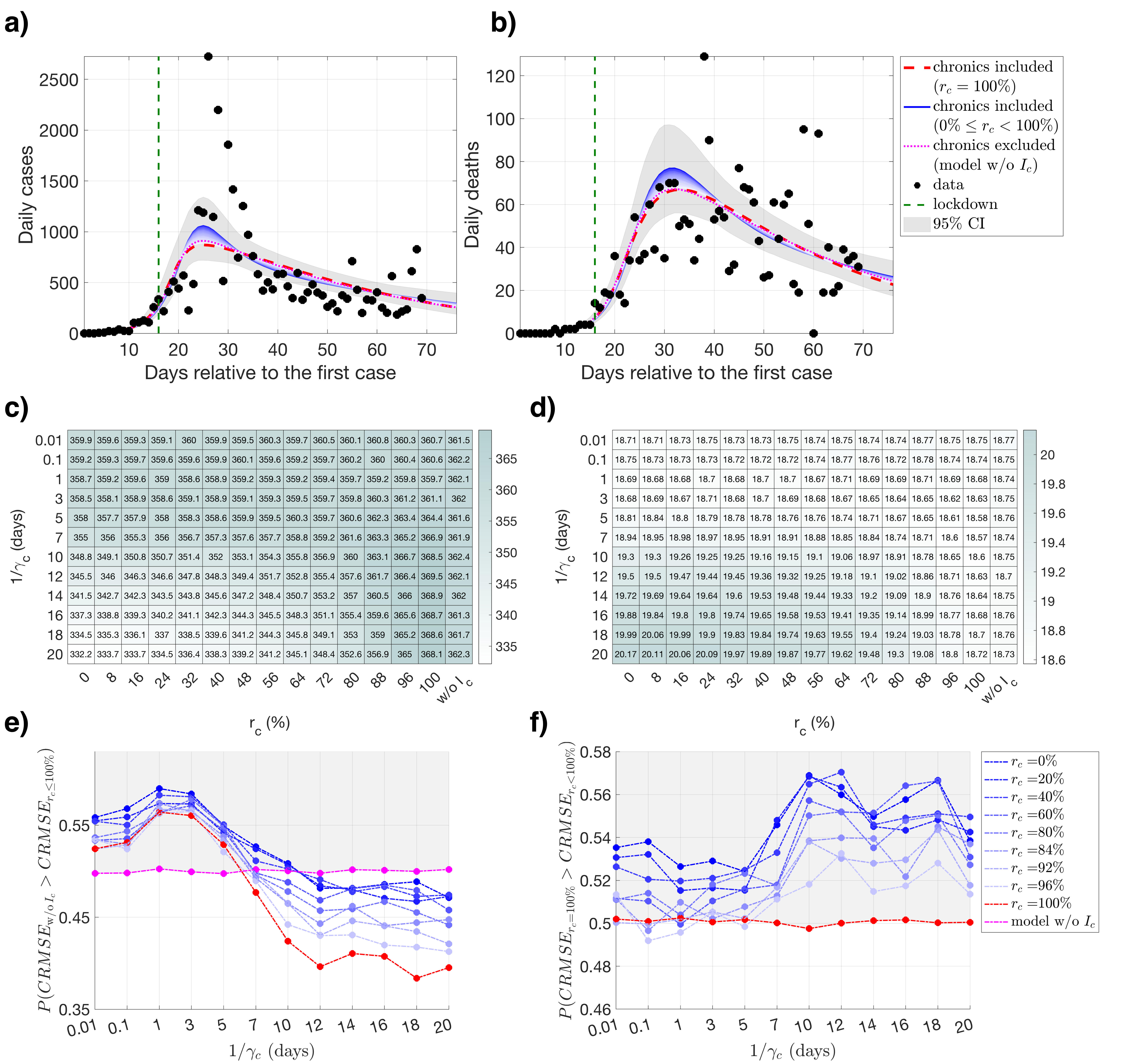

Supplement: S3 Fig — Fitting and RMSE results for the State of Louisiana, calculated using different levels and durations of infectiousness for the chronically infected population. Model outcomes (presented only for 1/γc = 14 days) for the number of a) daily confimed cases and b) daily deaths using the data until the introduction of relaxation for model fitting, respectively. Darker shades of blue represent the fitting results with increased infectiousness of the chronically infected population, i.e., lower rc values within the range 0 ≤ rc < 100%. Fitting results for rc = 100% are drawn in red, and the fitting results for the model without the Ic compartment (model w/o Ic) are drawn in pink. Data points that are used for fitting are drawn in black. Gray areas around the model outcomes represent the union of the 95% confidence intervals calculated for all models. RMSE values c) for the number of daily confirmed cases and d) the number of daily deaths for a given rc and γc value used for fitting, where model w/o Ic represents the results for the model without the Ic compartment. e) Probability of the model without the Ic compartment (model w/o Ic) having a greater combined RMSE (CRMSE) value than the model with the Ic compartment for all levels of reduced infectiousness (rc ≤ 100%) for different rc and γc values. f) Probability of the model where individuals are being diagnosed without being infectious (rc = 100%) having a greater combined RMSE (CRMSE) value than the model with individuals with a a prolonged infectiousness (rc < 100%) for different rc and γc values. Points in the gray areas represent the models that are providing a better fit more frequently than e) the model without the Ic compartment (model w/o Ic) and f) the model with rc = 100%. (TIFF) [file pcbi.1008609.s003.tiff]

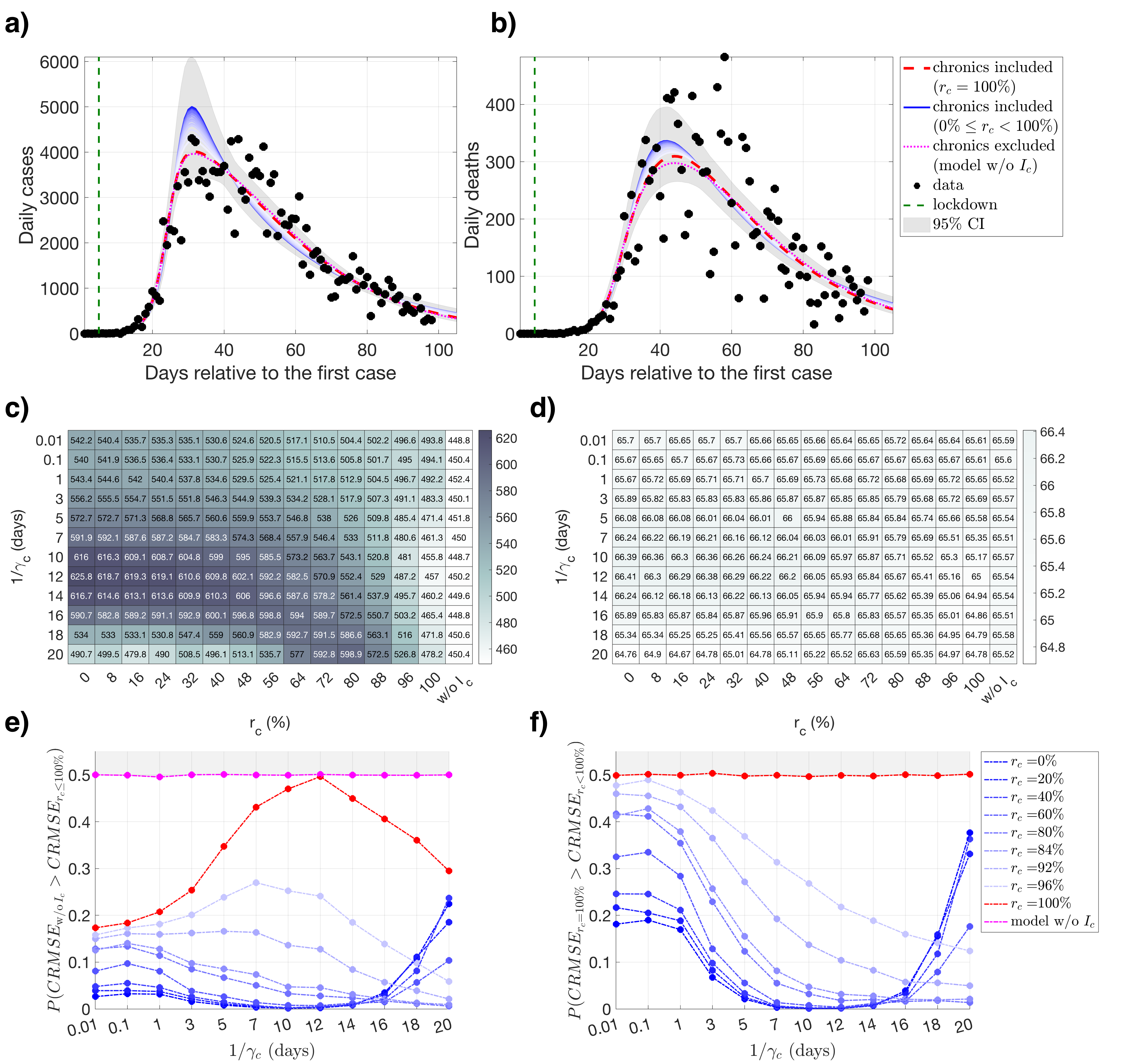

Supplement: S4 Fig — Fitting and RMSE results for the State of New Jersey, calculated using different levels and durations of infectiousness for the chronically infected population. Model outcomes (presented only for 1/γc = 14 days) for the number of a) daily confimed cases and b) daily deaths using the data until the introduction of relaxation for model fitting, respectively. Darker shades of blue represent the fitting results with increased infectiousness of the chronically infected population, i.e., lower rc values within the range 0 ≤ rc < 100%. Fitting results for rc = 100% are drawn in red, and the fitting results for the model without the Ic compartment (model w/o Ic) are drawn in pink. Data points that are used for fitting are drawn in black. Gray areas around the model outcomes represent the union of the 95% confidence intervals calculated for all models. RMSE values c) for the number of daily confirmed cases and d) the number of daily deaths for a given rc and γc value used for fitting, where model w/o Ic represents the results for the model without the Ic compartment. e) Probability of the model without the Ic compartment (model w/o Ic) having a greater combined RMSE (CRMSE) value than the model with the Ic compartment for all levels of reduced infectiousness (rc ≤ 100%) for different rc and γc values. f) Probability of the model where individuals are being diagnosed without being infectious (rc = 100%) having a greater combined RMSE (CRMSE) value than the model with individuals with a a prolonged infectiousness (rc < 100%) for different rc and γc values. Points in the gray areas represent the models that are providing a better fit more frequently than e) the model without the Ic compartment (model w/o Ic) and f) the model with rc = 100%. (TIFF) [file pcbi.1008609.s004.tiff]

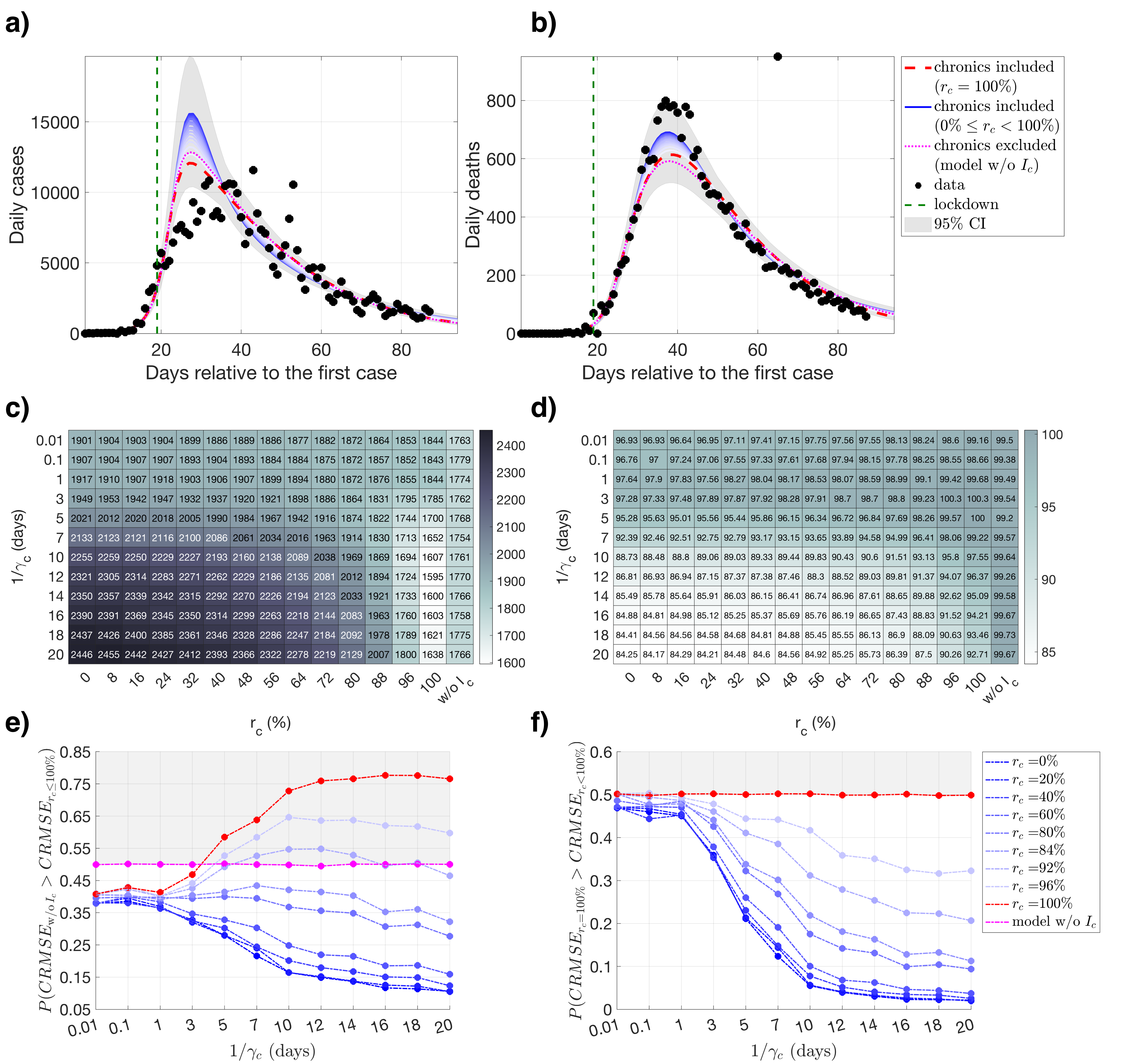

Supplement: S5 Fig — Fitting and RMSE results for the State of New York, calculated using different levels and durations of infectiousness for the chronically infected population. Model outcomes (presented only for 1/γc = 14 days) for the number of a) daily confimed cases and b) daily deaths using the data until the introduction of relaxation for model fitting, respectively. Darker shades of blue represent the fitting results with increased infectiousness of the chronically infected population, i.e., lower rc values within the range 0 ≤ rc < 100%. Fitting results for rc = 100% are drawn in red, and the fitting results for the model without the Ic compartment (model w/o Ic) are drawn in pink. Data points that are used for fitting are drawn in black. Gray areas around the model outcomes represent the union of the 95% confidence intervals calculated for all models. RMSE values c) for the number of daily confirmed cases and d) the number of daily deaths for a given rc and γc value used for fitting, where model w/o Ic represents the results for the model without the Ic compartment. e) Probability of the model without the Ic compartment (model w/o Ic) having a greater combined RMSE (CRMSE) value than the model with the Ic compartment for all levels of reduced infectiousness (rc ≤ 100%) for different rc and γc values. f) Probability of the model where individuals are being diagnosed without being infectious (rc = 100%) having a greater combined RMSE (CRMSE) value than the model with individuals with a a prolonged infectiousness (rc < 100%) for different rc and γc values. Points in the gray areas represent the models that are providing a better fit more frequently than e) the model without the Ic compartment (model w/o Ic) and f) the model with rc = 100%. (TIFF) [file pcbi.1008609.s005.tiff]

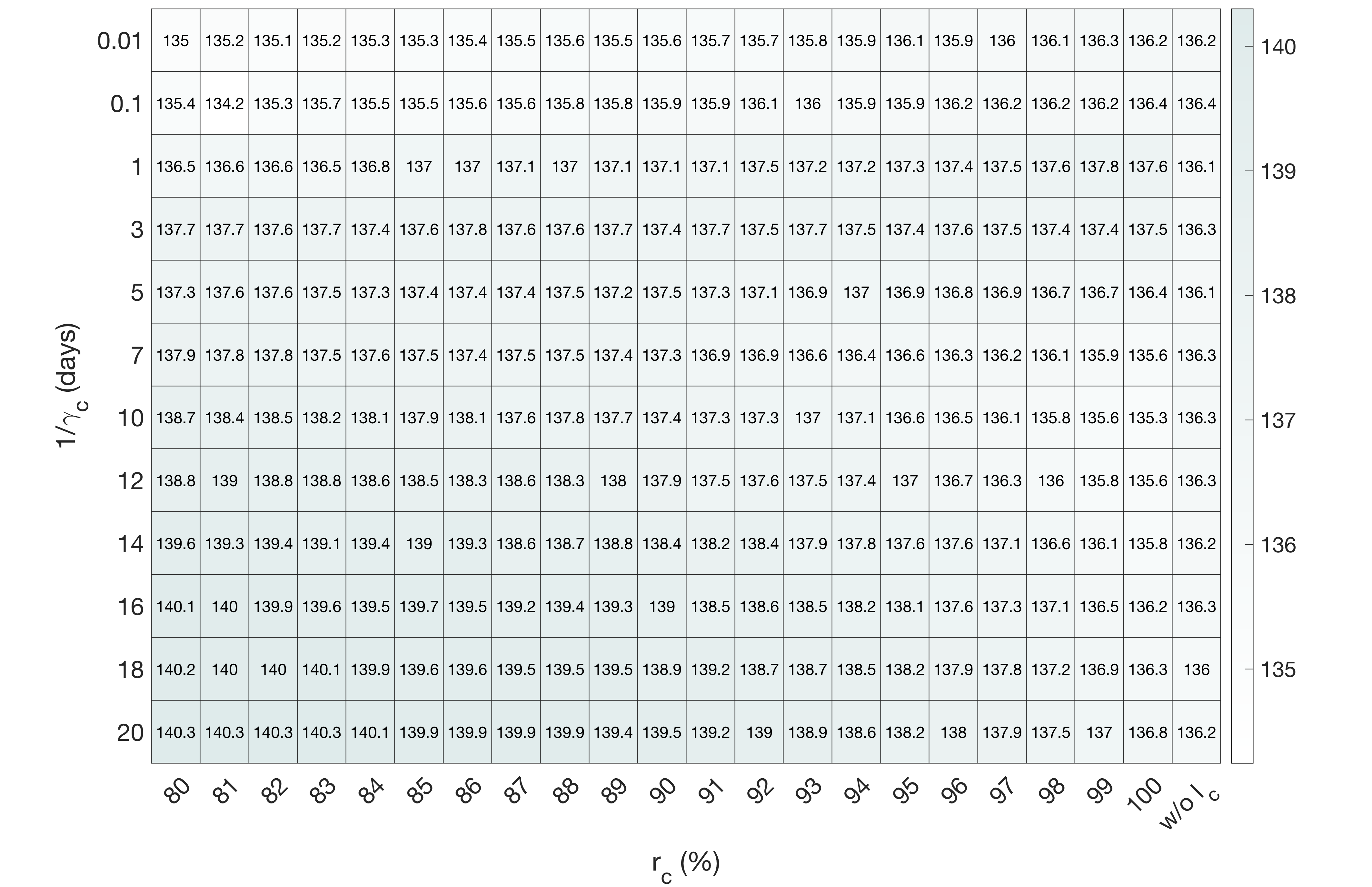

Supplement: S6 Fig — RMSE results of model fitting prior to the introduction of relaxation for Switzerland, calculated using different levels and durations of infectiousness for the chronically infected population. (TIFF) [file pcbi.1008609.s006.tiff]

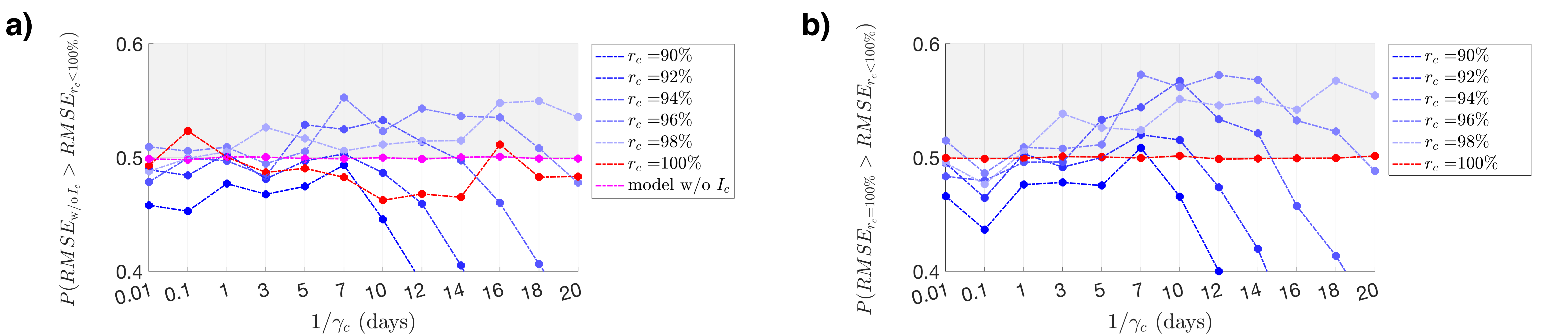

Supplement: S7 Fig — a) Probability of the model without the Ic compartment (model w/o Ic) having a greater RMSE value than the model with the Ic compartment for different levels of reduced infectiousness (90%≤rc ≤ 100%) and γc values, calculated over the predicted data points. b) Probability of the model where individuals are being diagnosed without being infectious (rc = 100%) having a greater RMSE value than the model with individuals with a a prolonged infectiousness (rc < 100%) for different rc and γc values, calculated over the predicted data points. Points in the gray areas represent the models that are providing a better fit more frequently than a) the model without the Ic compartment (model w/o Ic) and b) the model with rc = 100%. (TIFF) [file pcbi.1008609.s007.tiff]
